# Uterine transplantation: a systematic review

**DOI:** 10.6061/clinics/2016(11)10

**Published:** 2016-11

**Authors:** Dani Ejzenberg, Luana Regina Baratelli Carelli Mendes, Luciana Bertocco de Paiva Haddad, Edmund Chada Baracat, Luiz Augusto Carneiro D’Albuquerque, Wellington Andraus

**Affiliations:** I Departamento de Ginecologia, Faculdade de Medicina da Universidade de São Paulo, São Paulo/SP, Brasil; II Divisão de transplante de Órgãos Digestivos, Faculdade de Medicina da Universidade de São Paulo, São Paulo/SP, Brasil

**Keywords:** Infertility, Pregnancy, Uterus, Transplantation, Human

## Abstract

Up to 15% of the reproductive population is infertile, and 3 to 5% of these cases are caused by uterine dysfunction. This abnormality generally leads women to consider surrogacy or adoption. Uterine transplantation, although still experimental, may be an option in these cases. This systematic review will outline the recommendations, surgical aspects, immunosuppressive drugs and reproductive aspects related to experimental uterine transplantation in women.

## INTRODUCTION

Up to 15% of the reproductive population is infertile, and 3 to 5% of all cases of infertility are caused by uterine dysfunction 1. This abnormality generally leads women to consider surrogacy or adoption. However, in many countries, such as Japan and Sweden, surrogacy is heavily restricted or even prohibited. Uterine transplantation, although still experimental, may be an option in these cases 2.

The first pelvic organ transplantation, wich was performed in the 1960s, involved fallopian tube transplantation for the treatment of tube-peritoneal infertility, although this technique was only successful in an animal model 3. The first experiments in uterine transplantation were based on the premise that vascularization is improved if the uterus is transplanted in combination with the fallopian tubes. The first experimental model of uterine transplantation, published in 1973, was established in dogs 4,5, although the immunosuppressive drugs available at the time (cortisone and azathioprine) were inadequate to prevent rejection. The development of *in vitro* fertilization then diminished interest in fallopian tube transplantation, although infertility caused by uterine factors remained an issue. In this context, several animal models were studied, such as mice 6, rats 7, rabbits 8, sheep 9 and primates 10, predominantly in the early 1970s. The first human uterine transplantation was performed in 2002 in Saudi Arabia but resulted in graft loss and hysterectomy 3 months after transplantation 11. The second attempt at uterine transplantation occurred in Turkey in 2011 and resulted in two pregnancies, both ending in miscarriage 12. The first case with successful childbirth was reported in September 2014 among nine transplant patients in Gothenburg, Sweden 13. The 35-year-old patient had uterine agenesis, only one kidney and vaginal aplasia (a type of Rokitansky syndrome) and had received a uterus from a 61-year-old living donor 7 years past menopause. In November 2014, two more patients from the same study gave birth. The present systematic review will address the recommendations, surgical implications, immunosuppressive strategies and reproductive aspects related to experimental uterine transplantation in women.

## METHODOLOGY

This review of uterine transplantation was conducted in accordance with the PRISMA guidelines for systematic reviews 14. Multiple databases were searched, including Medline (PubMed), LILACS and the Cochrane Database. There were no restrictions on date, but the study was limited to English-language publications on human subjects.

**Figure 1:**
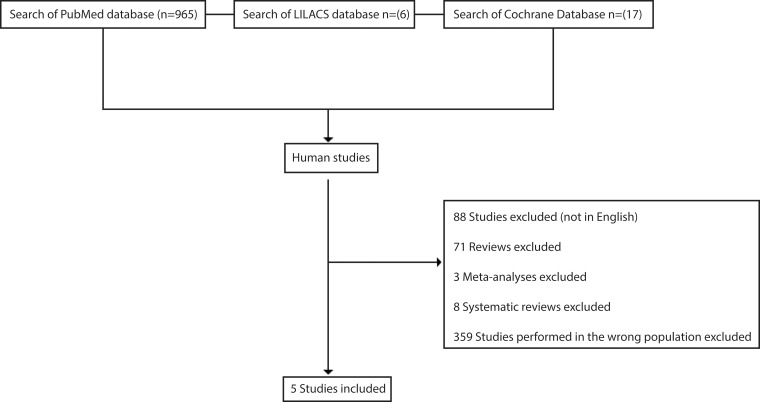
Literature flow diagram for the searches of the PubMed, LILACS and Cochrane databases.

The titles and abstracts of identified citations were screened for relevance by two independent reviewers. The full texts of “relevant” and “potentially relevant” articles were retrieved and evaluated independently by both reviewers. From each of the selected studies, the two reviewers independently extracted data on the year and journal of publication. Disagreements regarding the extracted data were solved by consensus or through consultation with a third reviewer. The characteristics of the selected articles were summarized and evaluated using narrative synthesis (Figure 1). The systematic literature analysis was specifically conducted using PubMed, LILACS and the Cochrane Database. In PubMed, a search for the terms (“Uterus” [Mesh]) AND (“transplantation" [Mesh]) yielded 965 studies, with 534 in human subjects. In LILACS, 6 articles were found following a search for the terms “uterus AND transplantation AND human”, but they were classified by the reviewers as irrelevant. Finally, in the Cochrane Database, 17 articles were found following a search for the terms “uterus AND transplantation AND human”, but these articles were also classified as irrelevant.

The articles that were included describe uterine transplantation techniques in humans with infertility caused by uterine dysfunction. Five articles were included in total.

### Uterus transplantation

#### Indications

Uterine transplantation may be recommended for women who wish to conceive who have uterine malformation or who have undergone previous hysterectomy due to neoplasm, post-partum hemorrhage, increased uterine bleeding or intrauterine adherences (Asherman syndrome). Uterine malformation affects up to 5% of the infertile population, with uterine agenesis and uterine hypoplasia being the most frequent causes. In particular, uterine agenesis affects 1 in every 4,500 women 15.

Hysterectomies are typically carried out because of myoma, adenomyosis or post-partum hemorrhage. In the USA, half of the patients attending a particular program for surrogacy had undergone hysterectomy. Moreover, malignant neoplasm of the uterine cervix is the third most common cancer in women. This cancer was responsible for over 5,000 deaths in 2010 in Brazil 16, and the Brazilian Institute of Cancer (INCA) estimates that there will be 15,590 new cases in 2015 17.

### Aspects of assisted reproduction

In a Swedish study of ovarian stimulation, a patient underwent nasal administration of a gonadotropin-releasing hormone agonist 9 days after high levels of luteinizing hormone were detected 13.

Follicular development was monitored through serial ultrasounds and serum estradiol measurements. During the first cycle of ovulation stimulation, only 150 IU of urinary gonadotropin was used. During the second and third cycles, 180 IU of recombinant gonadotropin and 225 IU of urinary gonadotropin were used, respectively. The duration of the cycles was approximately 11 to 14 days. Ovulation was triggered with human chorionic gonadotropin (HCG), and oocyte retrieval was subsequently performed. In the first cycle, only one egg cell was obtained, and one embryo was frozen. In the second cycle, 4 embryos were obtained from 9 eggs, and 6 embryos were obtained from 8 eggs in the third cycle. The first menstruation occurred 43 days after transplantation, and a single embryo was transferred approximately 12 months after transplantation, during the natural menstrual cycle, with ultrasound guidance 13.

### Aspects related to gestational and post-partum periods

The first successful pregnancy lacked complications until the 31^st^ week of gestation, when the patient presented a clinical condition similar to pre-eclampsia (hypertension, proteinuria and headaches). The patient was hospitalized and submitted to cesarean section due to alteration in fetal wellbeing. During the pregnancy, the patient took tacrolimus, azathioprine and prednisolone and presented a single mild rejection episode in the second month of gestation. The baby weighed 1775 g and was 40 cm in height at birth and was hospitalized for 16 days in the ICU. At discharge from the hospital, the baby weighed 2040 g 13. The two subsequent births reported occurred without complications 18.

### Surgical aspects

Donor Surgery - The donor can be living or deceased. The use of living donors was described by Brännström M. et al. in 2014 19. Using an infraumbilical midline incision, the uterus is dissected and isolated along with the ligaments that are important to hold it in the right position after implantation. A bilateral salpingectomy is also performed. The uterine arteries and veins are carefully dissected and separated from the ureters. The vagina is sectioned such that enough length is available for anastomosis in the recipient (10 to 15 mm). Additionally, the uterine vessel branches are cut along with a small patch from the iliac vessels, and soon after, the organ is perfused with preservative solution during the back-table procedure 20. Deceased donors are typically multiple organ donors, leading to a large midline incision and thus improved exposition. As a result, retrieval of the organ can be less restrictive, bringing more vessels with it (namely, iliac vessels), which can facilitate vascular reconstruction. The uterus is also heparinized and perfused with preservative solution before retrieval.

Recipient Surgery - Access is obtained through a midline infraumbilical incision. The iliac vessels (internal and external) are also dissected and isolated. It is important to synchronize the donor/recipient procedures to minimize cold ischemia time. Implantation is also different depending on the type of donor. In both cases, the native uterus is removed if present, and the space is prepared to receive the new organ (vagina, fixation points). In living donors, the implantation is as described by Brännström M. et al. 19, requiring more vessel anastomosis; in particular, arteries and veins are anastomosed on both sides (right and left) of the external iliac vessels. Deceased donation can allow less vessel anastomosis; the internal iliac vessels can be reconstructed during the back-table procedure and anastomosed via one artery (aorta or iliac extern) and one vein (cava or iliac extern). The vagina is then sutured, and the uterus is fixed (round and sacrouterine ligaments). The flow of the anastomosed vessels can be verified by ultrasound or fluxometry 19,20.

### Immunosuppression

Immunosuppressive therapy is continued until birth, when a cesarean section is performed and the allograft uterus is removed. For that reason, the complications that this therapy may cause for fetal development must be clarified. The only information on the safety of new drugs during pregnancy and lactation is from experimental and preclinical animal studies because experimental trials on pregnant or lactating mothers are prohibited. In the absence of controlled studies, negative reports have predominantly stemmed from pharmacovigilance, case reports and small case series 21. Table 1 shows the protocols used for immunosuppressive therapy in human uterine transplantation, involving induction and maintenance.

According to a systematic review of pregnancy in liver and kidney transplantation recipients 22, approximately 75% of all pregnant women who underwent liver or kidney transplantation had live births; this result may have been due to the special care provided to such patients by transplantation centers. Major congenital malformations were observed in approximately 3% of all transplanted pregnant women, a rate similar to that in the non-transplant population. Therefore, the majority of pregnancies in transplantation recipients are safe and uncomplicated. The safety of immunosuppressant drug use during pregnancy in transplanted women according to the Food and Drug Administration (FDA) classification is shown in Table 2.

## DISCUSSION

A significant body of global knowledge on solid organ transplantation has now been acquired and validated, establishing fertile grounds for the development of new surgical procedures. Better surgical technology and better understanding of immunosuppressive therapy, preventing severe adverse side effects, are factors contributing to this improvement.

Although only few cases of successful uterine transplantation have been reported, these cases represent a huge advance in the field of reproductive medicine, allowing pregnancy in patients with no other alternative.

This treatment belongs to a new category of transplantation surgeries that aim to improve patients’ quality of life, alongside procedures such as face or hand transplantation 23. The World Health Organization guarantees the right of women to procreate, and uterine transplantation is recommended for women who have uterine agenesis or a rudimentary uterus or who have undergone hysterectomy due to cervical, endometrial or ovary neoplasm; leiomyoma; or hemorrhage complications during delivery. Despite initial concern about the teratogenic effects of immunosuppression, the strategy implemented in the Swedish study was successful, so this strategy should be followed by other transplantation centers. In that study, the uterus was preserved post-partum, and immunosuppressive therapy was continued to attempt a second gestation in certain patients. Furthermore, the birth of healthy babies from organ-transplanted women has confirmed the efficacy and safety of certain immunosuppressive therapy during gestation following uterus transplantation 13,18,23,24.

The most successful cases of uterine transplantation involving live donors were reported in Gothenburg, Sweden 13. At the time of writing, no large series of transplantations using uteruses from deceased donors has been performed, and to date, none of the cases reported has resulted in a live birth. The lack of success with deceased donors could be related to the donors’ previous conditions, such as vasoactive drug use and elevated serum levels of inflammatory mediators, as well as to the type of preservative solution used to store the organ and the duration of cold ischemia. This situation can be improved via a better understanding of how to maintain the deceased donor and by both the development of a better preservative solution and the improvement of perfusion pumps. Therefore, the viability and potential success of transplantation performed with uteruses from deceased donors still need to be assessed.

Early tests of uterine transplantation have been much more promising than the early phases of transplantation of other solid organs, such as the kidney, heart and liver. However, the first uterine transplantation surgeries performed required a long time in surgery. In the future, following the completion of a larger number of cases and with the improvement of surgical techniques, this time will be reduced, as has been observed in the evolution of the transplantation of other organs.

In sum, uterine transplantation is a new and viable therapeutic option for patients with uterus-related infertility who wish to have a child, as long as this transplantation is performed at centers of expertise that specialize in human reproduction and transplantation techniques and related skills.

## AUTHOR CONTRIBUTIONS

Ejzenberg D, Mendes LR and Andraus W conceived and designed the study. Baracat EC and Carneiro D’Albuquerque LA reviewed the manuscript. Ejzenberg D, Mendes LR, Andraus W and Haddad LB were responsible for the analysis and interpretation. Ejzenberg D approved the final version of the manuscript.

## Figures and Tables

**Table 1:** Immunosuppressant therapy (induction, maintenance and rejection treatment) in human uterine transplantation studies.

Reference	Immunosuppression Scheme		
	Induction	Maintenance	Rejection Treatment
**Fageeh W. et al., 2002 (11)**	Cyclosporine 6 h prior to surgery and 500 mg IV methylprednisolone.	Cyclosporine, azathioprine and prednisolone.	Treated with cyclosporine, azathioprine, IV methylprednisolone and anti-thymocyte globulin.
**Erman Akar M. et al., 2013 (12)**	Anti-thymocyte globulin and 1 mg prednisolone.	TAC*, MMF** and prednisolone for the first 12 months. Then discontinued MMF and replaced with azathioprine.	Doses of prednisone and azathioprine were adjusted.
**Brännström M. et al., 2014 (19)**	1 g MMF, 500 mg methylprednisolone and anti-thymocyte globulin.	TAC, MMF and oral glucocorticosteroids once daily on the day of surgery and during the first 4 postoperative days.	Treated with corticosteroids.
**Brännström M. et al., 2015 (13)**	IV anti-thymocyte globulin just before surgery and 12 h later and 500 mg methylprednisolone.	TAC and MMF during the first 6 months. Withdrawal of MMF after 6 months and addition of azathioprine and prednisolone.	Treated with corticosteroids.
**Johannesson L. et al., 2015 (23)**	IV anti-thymocyte just before surgery and 12 h later and 500 mg methylprednisolone.	TAC and MMF during the first 10 months post-surgery. Azathioprine instead of MMF after 10 months to avoid the potentially teratogenic effects of MMF.	Five milligrams daily of prednisolone and corticosteroids or dose increments of TAC.

* *TAC* = tacrolimus,

** *MMF* = mycophenolate mofetil

**Table 2:** Safety of immunosuppressant drugs during pregnancy in transplanted women according to the FDA classification 22.

	FDA Safety Classification	Observation
**Steroids**	B – No evidence of risk in humans	No evidence of teratogenicity for steroids.
**Cyclosporine**	C – Risks cannot be ruled out	Does not lead to an increased rate of malformations but is associated with low birth weight.
**Tacrolimus**	C – Risks cannot be ruled out	Preterm birth, transient hyperkalemia and renal impairment.
**Everolimus/Sirolimus**	C – Risks cannot be ruled out	Limited knowledge is available on the use of mTOR inhibitors in pregnant women.
**Azathioprine**	D – Positive evidence of risk	Prematurity and low birth weight have been observed in pregnancies with azathioprine medication.
**MMF**	D – Positive evidence of risk	Strictly contraindicated in pregnancy and associated with miscarriage and a wide spectrum of malformations in the fetus.
